# Trans-Differentiation of Human Dental Pulp Stem Cells Into Cholinergic-Like Neurons Via Nerve Growth Factor

**DOI:** 10.32598/bcn.10.6.609

**Published:** 2019-11-01

**Authors:** Shahram Darabi, Taki Tiraihi, Maryam Nazm Bojnordi, Hatef Ghasemi Hamidabadi, Nourollah Rezaei, Maria Zahiri, Rafieh Alizadeh

**Affiliations:** 1. Cellular and Molecular Research Center, Qazvin University of Medical Science, Qazvin, Iran.; 2. Department of Anatomical Sciences, Faculty of Medical Sciences, Tarbiat Modares University, Tehran.; 3. Department of Anatomy & Cell Biology, Faculty of Medicine, Mazandaran University of Medical Sciences, Sari, Iran.; 4. Immunogenetic Research Center, Department of Anatomy & Cell Biology, Faculty of Medicine, Mazandaran University of Medical Sciences, Sari, Iran.; 5. The Persian Gulf Marine Biotechnology Research Center, The Persian Gulf Biomedical Sciences Research Institute, Bushehr University of Medical Sciences, Bushehr, Iran.; 6. Department of Anatomical Sciences, School of Medical Sciences, Bushehr University of Medical Sciences, Bushehr, Iran.; 7. ENT and Head & Neck Research Center and Department, Hazrat Rasoul Akram Hospital, Iran University of Medical Sciences, Tehran, Iran.

**Keywords:** Dental pulp, Stem cells, Cholinergic neurons, Differentiation, Nerve growth factor

## Abstract

**Introduction:**

Cell therapy has been widely considered as a therapeutic approach for neurodegenerative diseases and nervous system damage. Cholinergic neurons as one of the most important neurons that play a significant role in controlling emotions, mobility, and autonomic systems. In this study, Human Dental Pulp Stem Cells (hDPSCs) were differentiated into the cholinergic neurons by β-mercaptoethanol in the preinduction phase and also by the nerve growth factor (NGF) in the induction phase.

**Methods:**

The hDPSCs were evaluated for CD73, CD31, CD34, and Oct-4. Concentration-time relationships for NGF were assessed by evaluating the viability rate of cells and the immune response to nestin, neurofilament 160, microtubule-associated protein-2, and choline acetyltransferase.

**Results:**

The hDPSCs had a negative response to CD34 and CD31. The optimal dose for the NGF was 50 ng/mL seven days after the induction when the highest percentage of expressing markers for the Cholinergic neurons (ChAT) was detected.

**Conclusion:**

The results of this study provided a method for producing cholinergic neurons by hDPSCs, which can be used in cytotherapy for degenerative diseases of the nervous system and also spinal cord injury.

## Highlights

- Nerve growth factor increased differentiation of human dental pulp stem cells into cholinergic neurons.
- Human dental pulp stem cells were differentiated into the cholinergic neurons using βME.
- The optimal dose for nerve growth factor to induce cholinergic neural differentiation was 50 ng/mL.

## Plain Language Summary

Cell therapy is a therapeutic approach in neuroregenerative medicine. Cholinergic neurons have an essential role in emotions, mobility, and autonomic systems. Here, we used human dental pulp stem cells (hDPSCs) to produce cholinergic neurons using some growth factors, such as β-mercaptoethanol and nerve growth factor (NGF). We found that β-mercaptoethanol and NGF increased the differentiation of hDPSCs into cholinergic neurons. Also, the optimal dose for NGF to induce cholinergic neural differentiation was 50 ng/mL. The protocol of this study can be used in cytotherapy in degenerative diseases of the nervous system and spinal cord injury.

## 1. Introduction

Nowadays, cell therapy is highly regarded as one of the therapeutic methods for nervous system injuries (Naghdi et al., 2009; Darabi et al., 2013). Different types of cells, such as embryonic stem cells (ESCs), adult stem cells, and umbilical cord stem cells have been studied for transplantation into the nervous system (Boncoraglio et al., 2010; Bojnordi et al., 2012). The cholinergic neurons are used for the treatment of motor neuron degeneration (Abdanipour et al., 2014; Bojnordi et al., 2013) and Alzheimer disease (Thonhoff et al., 2009).

In previous studies, cholinergic neurons have been generated from ESCs and induced Pluripotent Stem Cells (iPSCs). Because of the allogeneic, tumorigenic, and ethical problems, using other mesenchymal stem cells has been suggested. However, Bone Marrow Stromal stem Cells (BMSCs) and Adipose-Derived Stem Cells (ADSCs) have been applied for neuronal differentiation (Darvishi et al., 2017; Naghdi et al., 2013; Nizzardo et al., 2010; Ronaghi et al., 2010; Alizadeh et al., 2017). Interestingly, Human Dental Pulp Stem Cells (hDPSCs) have a high capacity to differentiate into neurons. It has shown that transplantation of the cholinergic neurons into animal models could increase the survival rate of laboratory animals (Lee et al., 2014). Since the identification of hDPSCs by Gronthos et al., (2000), other researchers have investigated hDPSCs ability to differentiate into other cell lines (Karaöz et al., 2010; Arthur et al., 2008). Many studies have revealed DPSCs differentiation potential into neural cells in vitro (Kiraly et al., 2009; Chun et al., 2016). Moreover, after injection into the chicken and rat brain, hDPSCs can express neural markers and respond to brain neurotrophic factors (Király et al., 2011; Leong et al., 2012). Although in the neuronal culture medium, Mesenchymal Stem Cells (MSCs) are differentiated into neurons, astrocytes, and oligodendrocytes (Fu et al., 2008; Darabi et al., 2017; Alizadeh et al., 2017), their efficiency is very low and predictable. Recently, hDPSCs due to their embryonic origin have become a promising source for cell therapy (Fu et al., 2008).

The hDPSCs originate from neural crest cells and have neuronal specifications (Fu et al., 2008; Darabi et al., 2017; Alizadeh et al., 2015). They also are known as ectomesenchymal cellsderived from the ectoderm around the neural tube and migrate to the areas within the tooth and dental pulp leading to a mesenchymal phenotype. MSCs are not rejected by the immune system and present no ethical issues (Abbaszadeh et al., 2014; Haratizadeh et al., 2016). Therefore, these cells are suitable for cell therapy in nervous system diseases. In a study, due to the secretion of neuronal factors, Dental Pulp Stem Cells (DPSC) increased the survival of tyrosine hydroxylase neurons in the culture medium (Haratizadeh et al., 2016). In normal circumstances and without neural lineage induction, hDPSCs could express some neuronal factors, such as nestin and Glial Fibrillary Acidic Protein (GFAP) at the level of genes and proteins (English et al., 2014).

In the neural induction medium, hDPSCs can express the specific markers for post-mitotic mature neurons, like Neuronal Nuclear antigen (NeuN) (Le Blanc, 2006; Ronaghi et al., 2010). Although DPSC can become Neural-Like Cells (NLCs) in differentiation medium, it cannot be differentiated completely (Bojnordi et al., 2018). In previous studies on DPSC differentiation, 5% of hDPSCs has differentiated into steroid cells expressing GFAP (Nosrat et al., 2004). In another study, about 53% of choline acetyltransferase (ChAT)-positive motor neurons were generated from hDPSCs (Ellis et al., 2014).

It seems that the hDPSCs can differentiate into specific subtypes of neurons under appropriate conditions. The current research aimed to create ChAT-positive cells from hDPSCs during two phases of preinduction and induction by β-mercaptoethanol (βME) and Nerve Growth Factor (NGF), respectively.

## 2. Methods

### 2.1. Isolation and cultivation of hDPSCs

In this study, hDPSCs were isolated from the human third molar. Healthy teeth without cavities were collected from 18–25 years old patients referred to the dental clinic of the Mazandaran University of Medical Sciences. The informed consent was obtained from the participants, and the research was approved by the Human Ethics Committee of Mazandaran University of Medical Sciences. Teeth were washed in Phosphate-Buffered Saline (PBS). To disinfect the teeth, iodine (povidone-iodine 10%, Pejnan, Iran) was used for 5 min. Then, using a separator and surgery cutter, root and dentin were broken and pulp tissue was isolated by sterile forceps. Pulp tissue digestions were done by scalpel and trypsin 0.25% (Gibco, USA). Pulp tissues with trypsin and DMEM/F12 (Gibco-Life Technologies, Canada) were poured in a falcon tube and kept in an incubator for 5 min. In the next step, the falcon tube was centrifuged, the supernatant was removed and pulp tissues were transferred into cell culture flasks containing medium and incubated at 37° C with 5% CO _2_ and 95% humidity. The cell culture flask was examined every 2–3 days. After 3–4 passages, hDPSCs were used (Gronthos et al., 2000).

### 2.2. Differentiation of hDPSCs into neurons

For preinduction step, hDPSCs were seeded into 6-well plates. After 24 h, 1 mM of βME (Sigma) was added into the wells and kept for 48 h. At the induction phase, the optimal dose of NGF (R & D Systems, USA) was evaluated using a dose-response (1, 25, 50, and 100 ng/mL) and time-course (2 and 7 days) evaluation, in which the preinduced hDPSCs were incubated with this protocol. Morphological analysis of differentiating cells was assessed in different groups by an inverted microscope (Nikon, Eclipse-TS100), dimethylthiazolyl diphenyl tetrazolium bromide (MTT) assay, and immunocytochemistry technique.

### 2.3. MTT assay

To achieve the optimal dose of NGF, the viability of hDPSCs, preinduced, and induced cells were assessed by MTT assay. Briefly, the 96-well plates were incubated with MTT (5 mg/mL in PBS, Sigma-Aldrich) for 3 h at 37 °С. Formazan insoluble crystals were then solved in dimethyl sulfoxide (DMSO, Sigma). The absorbance of formazan products was determined by the ELISA plate reader (Bio Tek, USA) at 570 nm (Fu et al., 2008).

### 2.4. Immunocytochemistry

After the third passage, hDPSCs were assessed for Oct-4 (a pluripotency stem cell marker), CD73 (mesenchymal stromal cell marker), CD31 (endothelial cell marker), and CD34 (hematopoietic stem cells marker) by immunocytochemical assessment. Immunocytochemistry technique evaluated immunoreaction of hDPSCs, preinduced, and induced cells to nestin, neurofilament 160 (NF160), microtubule-associated protein 2 (MAP2), and ChAT.

Cells were washed three times with PBS for 5 min and then fixed in 4% paraformaldehyde (Sigma-Aldrich) for 15 min. Next, they were rewashed with PBS. In the next step, the cells were permeabilized with 1% Triton X-100 (Sigma-Aldrich) for 30 min and blocked with bovine serum albumin (Sigma-Aldrich) for 45 min. The cells were incubated with the rabbit anti-mouse secondary antibody conjugated with FITC (1:100; Millipore) for 2 h at room temperature. The cells were then rinsed twice in PBS for 15 min and counterstained with propidium iodide to visualize the nuclei. They were rewashed in PBS and examined using a fluorescence microscope (Nikon, Eclipse-TE600, Japan). All antibodies were prepared in mouse, and the following dilutions were used as follows: mouse anti-CD31 monoclonal antibody (1:200; Millipore), mouse anti-CD73 monoclonal antibody (1:300; Millipore), mouse anti-CD34 monoclonal antibody (1:300; Millipore), mouse anti-Oct4 monoclonal antibody (1:400), mouse anti-nestin monoclonal antibody (1:300; Millipore), mouse anti-neurofilament 160 kDa (anti-NF160) monoclonal antibody (1:300; Millipore), mouse anti-MAP2 monoclonal antibody (1:500, Abcam system), and mouse anti-ChAT monoclonal antibody (1:500; Abcam system).

The number of immunopositive cells for neuronal markers was obtained using the captures of immunostained cultures, counted, and then divided by the total number of cells (propidium iodide-stained cells) in at least five random nonoverlapping selected fields, where 500 cells were counted in three independent cultures. The results were expressed as Mean±SD.

### 2.5. Statistical analysis

One-way Analysis of Variance (ANOVA) and Tukey’s posthoc test were used for data analysis in SPSS V. 13.0. P-values of less than 0.05 were considered significant.

## 3. Results

### 3.1. hDPSCs culture and characterization

**Figure 1.**
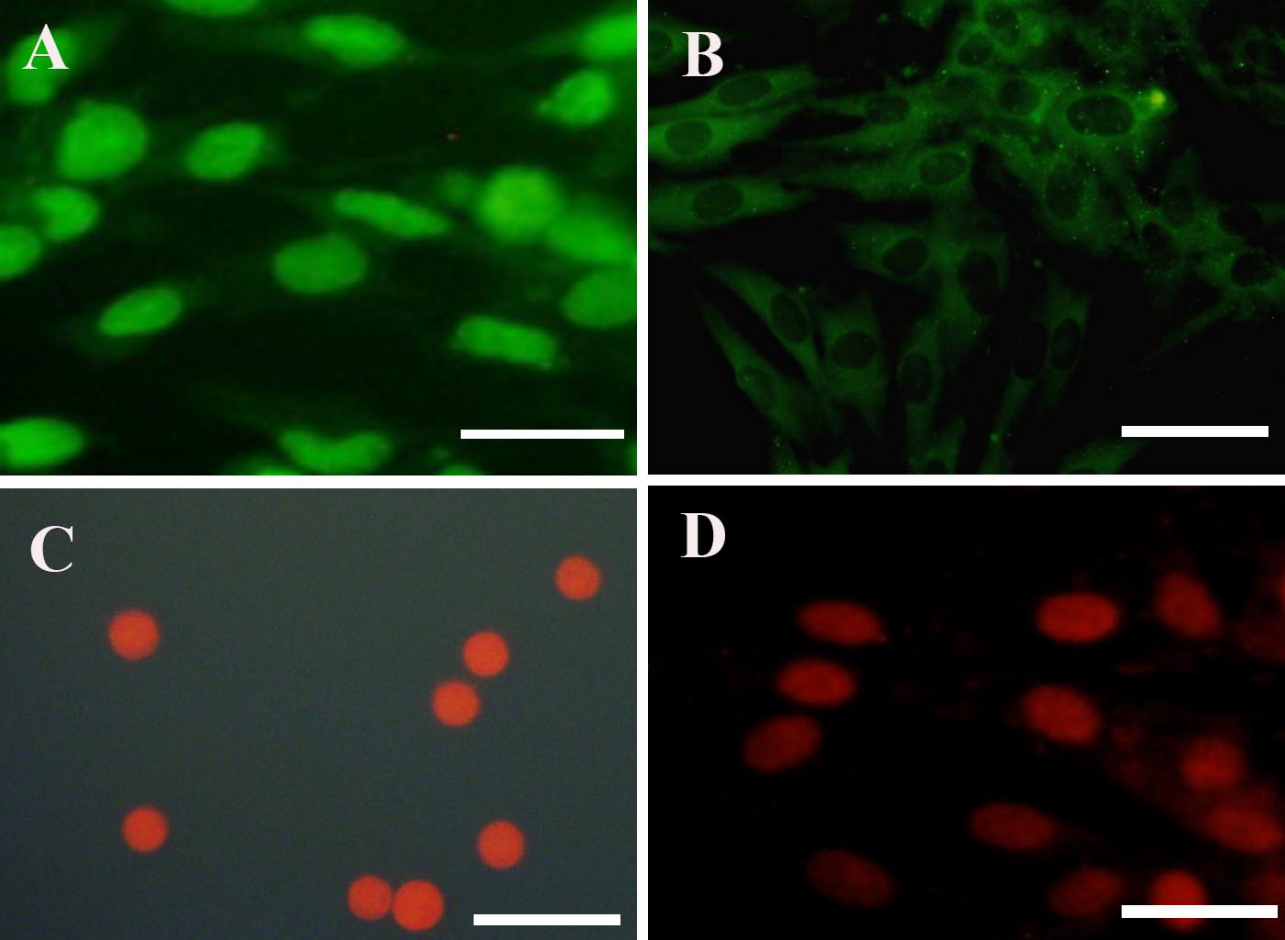
Immunostaining of cultured hDPSCs A, B, C, and D. represent immunostaining of Oct-4, CD73, CD31, and CD34, respectively. hDPSCs were immunolabeled with the primary antibody, incubated with FITC-conjugated secondary antibody, and counter-stained using propidium iodide (Scale bars: 50 μm). hDPSCs: Human Dental Pulp Stem Cells

After enzymatic digestion, hDPSCs were plated onto a T25 culture flask. The cells were attached to the bottom of the flask and had a uniform population of fibroblast-like cell bodies and appendages. hDPSCs were strongly positive for Oct-4 as a stem cell pluripotency marker and CD73 as a mesenchymal stromal cell marker. However, they were negative for CD31 as an endothelial cell marker and CD34 as a hematopoietic stem cell marker (Figure 1).

### 3.1. hDPSCs differentiation

**Figure 2.**
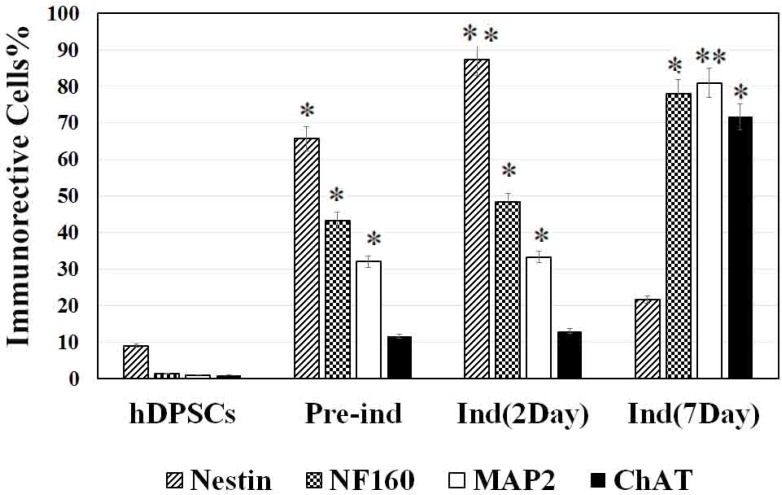
A histogram indicating the dose-response experiment The dose-response human dental pulp stem cells-derived cholinergic-like neurons viability at different concentrations of NGF (1, 25, 50, and 100 ng/mL) after 7 days detected by MTT assay. Each column represents the average measurement from five replicates. The asterisk shows significant differences compared with the other groups (P<0.05). NGF: Nerve Growth Factor; MTT: Dimethylthiazolyl Diphenyl Tetrazolium Bromide

To find out the optimal dose and induction time of NGF, the preinduced cells were exposed to various concentrations of NGF (1, 25, 50, and 100 ng/mL) for 7 days (Figure 2). Then, the cell viability was investigated 7 days after NGF treatment. The results of the MTT assay showed that the viability of the cells at 100 ng/mL concentration was significantly lower than other doses (P<0.05) (Figure 2).

**Figure 3.**
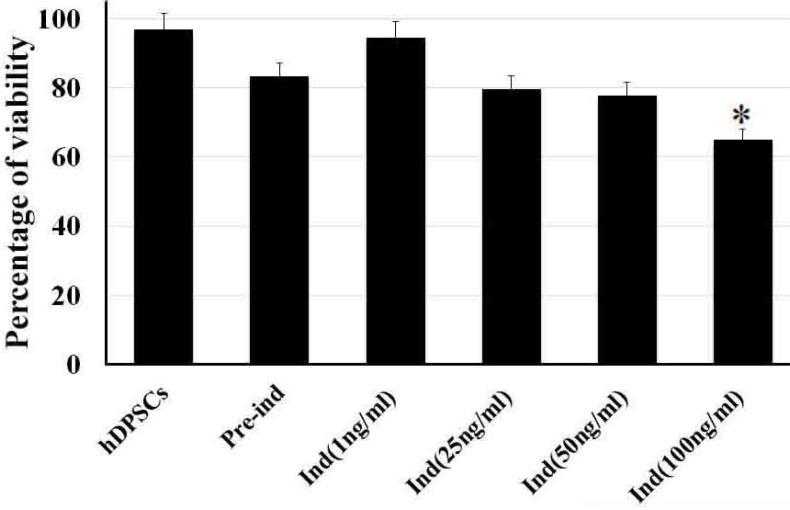
Histogram of the mean percentages of the immunoreactive cells to nestin NF160, MAP2, and ChAT (Hatched lines pattern, chessboard pattern, white and black solid columns, respectively) of the hDPSCs, preinduced, and induced cells with 50 ng of NGF using the time-course evaluation (2 and 7 days). The asterisk shows significant differences compared with the control group (hDPSCs) with the same antibody (P<0.05). hDPSCs: Human Dental Pulp Stem Cells; NF 160: Neurofilament 160; MAP2: Microtubule-Associated Protein 2

Before hDPSCs induction, they expressed nestin (neural stem cells [NSCs] marker) by less than 10% (P<0.05). They also expressed other neuronal markers, such as NF160 (a neuronal intermediate filament marker), microtubule-associated protein 2 (MAP2a marker for neuronal differentiation), and ChAT (a cholinergic neuron marker) by less than 1% (Figure 2). Following preinduction with βME, the expression rate of nestin, NF160, MAP2, and ChAT increased (P<0.05; Figure 3).

The differentiation of hDPSCs into ChAT-positive cells was optimized at 50 ng/mL of NGF at the end of the seventh day (Figure 3) when the viability was recorded about 77.7% (Figure 2). On the seventh day of the induction phase, the expression rate of ChAT-positive cells increased to 71.66% compared with preinduction phase and the second day of induction, which was in favor of differentiation into cholinergic neurons (P<0.05; Figure 3). This result showed a significant difference in differentiation into cholinergic neurons.

**Figure 4.**
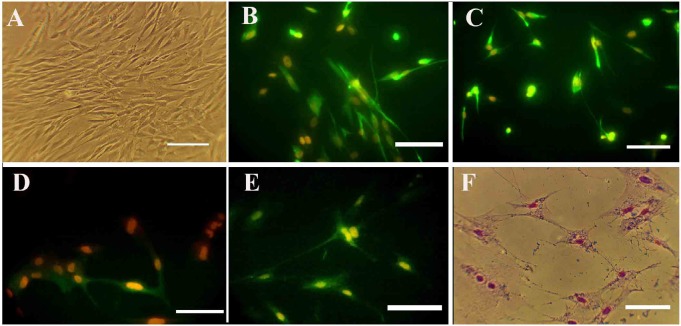
Photomicrographs of hDPSCs for specific neural markers A, B, C, D, E, and F. Represent the third passage, nestin, NF160, MAP2, ChAT, and cresyl violet staining, respectively. Nuclei were counterstained with propidium iodide (Scale bars: 50 μm). hDPSCs: Human Dental Pulp Stem Cells; NF 160: Neurofilament 160; MAP2: Microtubule-Associated Protein 2

The percentages of the induced cells were 21.66%, 78.81%, and 71.66% for nestin, NF160, MAP2, and ChAT, respectively (Figure 4). On the second and seventh days of induction, the cells were stained with cresyl fast violet, which showed the neural phenotype of the induced cells (Figure 4).

## 4. Discussion

Given the limited number of endogenous NSCs (Shi et al., 2011; Chang et al., 2014) and the inability of the central nervous system to repair, the generated cells can be a feasible source for replacement therapy (Shi et al., 2011; Chang et al., 2014; Sheng 2001; Moayeri et al., 2017, Chai et al., 2000). Two preinduction methods have been proposed to improve the differentiation process. The NF68 and nestin are expressed in the early stages of neuron differentiation, followed by MAP2 and NF160 (Carden et al., 1987). The results of using the βME in preinduction indicated that in this stage, the cells are neuroblasts (Naghdi et al., 2009; Lariviere, 2004). The MAP2 marker is located on both sides of the pre-synapse and postsynapse (Kitamura et al., 2007). The MAP2 expression profiles in preinduction indicated that the cells were not fully differentiated in this stage (Chiu et al., 1995) and were immature. The MAP2 and ChAT expression pattern, which were expressed significantly lower in the preinduction compared with the induction stage, is indicative of differentiation toward cholinergic-like neurons. The majority of neurons reduce the expression of the nestin during differentiation and maturation and increase the expression of neuronal markers, such as MAP2 and ChAT (Lu et al., 2004). Hung et al., found that induction of the BMSCs into a neuronal phenotype by βME is not stable (Aloe et al., 1986), and after a while, the cells revert to their original status.

However, in this study, the NGF was used as an inductor after 48 h of preinduction by βME. The expression levels of the ChAT reached from 10% in the preinduction to about 72% in the induction phase. However, the NGF is a strong neuronal protective, which can protect the cell damages against the βME. On the other hand, the βME prevents neurons from oxidative stress by increasing the levels of reduced glutathione, which in turn, increases the ChAT activity and growth of neuronal redundancies (Ni, Wen, Peng, & Jonakait, 2001). In an in vivo study, it was observed that the NGF enhanced expression of genes regulating the secretion of acetylcholine (Aloe et al., 1986).

After the third passage, the hDPSCs develop a fibroblast-like morphology. Immunohistochemical studies have shown that isolated cells are negative for hematopoietic cell markers (CD45/CD34) and CD106. These findings are consistent with the results of other studies (Hung et al., 2002). Several studies have demonstrated the role of hDPSCs in the healing of spinal cord injury (Nosrat et al., 2004; Ellis et al., 2014; Shi et al., 2011).

hDPSCs secrete NGF, glial cell-derived neurotrophic Factor (GDNF), Brain-Derived Neurotrophic Factor (BDNF), and BMP2 neurotrophic factors in the medium, which increase the lifespan of cells.

In another study, hDPSCs were transplanted to a rat model with severe spinal cord injury, and it was reported that hDPSCs increased axon growth by preventing apoptosis of neurons, astrocytes, and oligodendrocytes (de Almeida et al., 2011).

Exposure of hDPSCs to epidermal growth factor, basic fibroblast growth factor, and retinoic acid (Arthur et al., 2008) or forskolin (Kiraly et al., 2011), resulted in their differentiation into neural cells and expressed neuronal markers (Arthur et al., 2008). By injection of differentiated hDPSCs into the cerebrospinal fluid of rats, they integrated into the damaged brain parenchyma and survived for 4 weeks, where they expressed neuronal markers, including NeuN (Haratizadeh et al., 2016). The hDPSCs secrete the Vascular Endothelial Growth Factor (VEGF) and Platelet-Derived Growth Factor (PDGF) (Nosrat et al., 2004; Mead et al., 2014). The NGF plays a vital role in axons and neurons growth, whereas the BDNF and PDGF are essential factors for neuroprotection (Mead et al., 2014).

Based on transplantation studies, injection of hDPSCs into the healthy brain can stimulate the migration and proliferation of endogenous NSCs and increases the expression of the ciliary neurotrophic factor, VEGF, and FGF in the transplanted site (Hung et al., 2002). Although the graft has a very short lifespan, this study showed that the hDPSCs affect the adjacent cells through factors.

## 5. Conclusion

In an experimental model of Parkinson disease, hDPSCs were cultured and transplanted into experimental model of Parkinson. The death rate of neurons and cytotoxicity declined due to the expression of the NGF, GDNF, and BDNF by the hDPSCs (Leong et al., 2012). After the hDPSCs injection in the spinal cord injury, these cells did not differentiate into neurons but increased the lifespan of neurons and glia in the injury site and its surrounding environment (Sakai et al., 2012). Axons in the spinal cord injury grew underneath the lesion site in addition to repair the scar. This axonal growth was accompanied by improved motor function after the hDPSCs injection, which was more than other cells, such as the BMSCs and ADSCs (Soundararajan et al., 2007; Wichterle et al., 2002; Takahashi et al., 1999; Karaöz et al., 2011). The results of this study showed that using NGF, as an inducer, hDPSCs can differentiate into cholinergic neurons, which can be used for cytotherapy in degenerative diseases of the nervous system and spinal cord injury.

## Ethical Considerations

### Compliance with ethical guidelines

All ethical principles were considered in this article. The participants were informed about the purpose of the research and its implementation stages; they were also assured about the confidentiality of their information; Moreover, They were allowed to leave the study whenever they wish, and if desired, the results of the research would be available to them.

## References

[B1] Abbaszadeh H. A. Tiraihi T. Delshad A. Saghedizadeh M. Taheri T. Kazemi, (2014). Differentiation of neurosphere-derived rat neural stem cells into oligodendrocyte-like cells by repressing PDGF-α and Olig^2^ with triiodothyronine. Tissue and Cell, 46(6), 462–9. [DOI:10.1016/j.tice.2014.08.003] [PMID ] 25200619

[B2] Abdanipour A. Tiraihi T. Taheri T. (2014). Intraspinal transplantation of motoneuron-like cell combined with delivery of polymer-based glial cell line-derived neurotrophic factor for repair of spinal cord contusion injury. Neural Regeneration Research, 9(10), 1003–13. [DOI:10.4103/1673-5374.133159] [PMID ] [PMCID] 25206752 PMC4146307

[B3] Alizadeh R. Hassanzadeh G. Joghataei M. T. Soleimani M. Moradi F. Mohammadpour S. (2017). In vitro differentiation of neural stem cells derived from human olfactory bulb into dopaminergic-like neurons. European Journal of Neuroscience, 45(6), 773–84. [DOI:10.1111/ejn.13504] [PMID] 27987378

[B4] Alizadeh R. Hassanzadeh G. Soleimani M. Joghataei M. T. Siavashi V. Khorgami Z., (2015). Gender and age related changes in number of dopaminergic neurons in adult human olfactory bulb. Journal of chemical neuroanatomy, 69, 1–6. [DOI:10.1016/j.jchemneu.2015.07.003] [PMID ] 26212581

[B5] Aloe L. Alleva E. Böhm A. Levi-Montalcini R. (1986). Aggressive behavior induces release of nerve growth factor from mouse salivary gland into the bloodstream. Proceedings of the National Academy of Sciences, 83(16), 6184–7. [DOI:10.1073/pnas.83.16.6184] [PMID ] [PMCID ] PMC386464 3090553

[B6] Arthur A. Rychkov G. Shi S. Koblar S. A. Gronthos S. (2008). Adult human dental pulp stem cells differentiate toward functionally active neurons under appropriate environmental cues. Stem Cells, 26(7), 1787–95. [DOI:10.1634/stemcells.2007-0979] [PMID ] 18499892

[B7] Bojnordi M. N. Ebrahimi-Barough S. Vojoudi E. Hamidabadi H. G. (2018). Silk nanofibrous electrospun scaffold enhances differentiation of embryonic stem like cells derived from testis in to mature neuron. Journal of Biomedical Materials Research Part A, 106(10), 2662–9. [DOI:10.1002/jbm.a.36463] [PMID ] 29901281

[B8] Bojnordi M. N. Movahedin M. Tiraihi T. Javan M. (2012). A simple co-culture system for generation of embryonic stem-like cells from testis. Iranian Red Crescent Medical Journal, 14(12), 811. [DOI:10.5812/ircmj.4051] [PMID ] [PMCID ] 23483704 PMC3587872

[B9] Bojnordi M. N. Movahedin M. Tiraihi T. Javan M. (2013). Alteration in genes expression patterns during in vitro differentiation of mouse spermatogonial cells into neuroepithelial-like cells. Cytotechnology, 65(1), 97–104. [DOI:10.1007/s10616-012-9465-y] [PMID ] [PMCID ] 23104269 PMC3536871

[B10] Boncoraglio G. B. Bersano A. Candelise L. Reynolds B. A. Parati E. A. (2010). Stem cell transplantation for ischemic stroke. Cochrane Database of Systematic Reviews, 9, CD007231. [DOI:10.1002/14651858.CD007231.pub2] [PMID ] 20824857

[B11] Carden M. J. Trojanowski J. Q. Schlaepfer W. W. Lee V. M. (1987). Two-stage expression of neurofilament polypeptides during rat neurogenesis with early establishment of adult phosphorylation patterns. Journal of Neuroscience, 7(11), 3489–504. [DOI:10.1523/JNEUROSCI.07-11-03489.1987] [PMCID ] 3119790 PMC6569049

[B12] Chai Y. Jiang X. Ito Y. Bringas P. Han J. Rowitch D.H., (2000). Fate of the mammalian cranial neural crest during tooth and mandibular morphogenesis. Development, 127(8), 1671–9. [PMID ] 10725243 10.1242/dev.127.8.1671

[B13] Chang C. C. Chang K. C. Tsai S. J. Chang H. H. Lin C. P. (2014). Neurogenic differentiation of dental pulp stem cells to neuron-like cells in dopaminergic and motor neuronal inductive media. Journal of the Formosan Medical Association, 113(12), 956–65. [DOI:10.1016/j.jfma.2014.09.003] [PMID ] 25438878

[B14] Chiu F. C. Feng L. Chan S. O. Padin C. Federoff H. J. (1995). Expression of neurofilament proteins during retinoic acid-induced differentiation of P19 embryonal carcinoma cells. Molecular Brain Research, 30(1), 77–86. [DOI:10.1016/0169-328X(94)00280-R] 7609647

[B15] Chun S. Y. Soker S. Jang Y. J. Kwon T. G. E. S. (2016). Differentiation of human dental pulp stem cells into dopaminergic neuron-like cells in vitro. Yoo Journal of Korean Medical Science, 31(2), 171–7. [DOI:10.3346/jkms.2016.31.2.171] [PMID ] [PMCID ] 26839468 PMC4729494

[B16] Darabi S. Tiraihi T. Delshad A. Sadeghizadeh M. Taheri T. Hassoun H. K. (2017). Creatine enhances transdifferentiation of bone marrow stromal cell-derived neural stem cell into gabaergic neuron-like cells characterized with differential gene expression. Molecular Neurobiology, 54(3), 1978–91. [DOI:10.1007/s12035-016-9782-9] [PMID ] 26910814

[B17] Darabi S. Tiraihi T. Ruintan A. Abbaszadeh H. A. Delshad A. Taheri T. (2013). Polarized neural stem cells derived from adult bone marrow stromal cells develop a rosette-like structure. In Vitro Cellular & Developmental Biology-Animal, 49(8), 638–52. [DOI:10.1007/s11626-013-9628-y] [PMID ] 23771792

[B18] Darvishi M. Tiraihi T. Mesbah-Namin S. A. Delshad A. Taheri T. (2017). Motor neuron transdifferentiation of neural stem cell from adipose-derived stem cell characterized by differential gene expression. Cellular and Molecular Neurobiology, 37(2), 275–89. [DOI:10.1007/s10571-016-0368-x] [PMID ] 27107758 PMC11482063

[B19] de Almeida F. M. Marques S. A. Ramalho B. D. S. Rodrigues R. F. Cadilhe D. V. Furtado D. (2011). Human dental pulp cells: A new source of cell therapy in a mouse model of compressive spinal cord injury. Journal of Neurotrauma. 28(9), 1939–49. [DOI:10.1089/neu.2010.1317] [PMID ] 21609310

[B20] Ellis K. M. O’Carroll D. C. Lewis M. D. Rychkov G. Y. Koblar S. A. (2014). Neurogenic potential of dental pulp stem cells isolated from murine incisors. Stem Cell Research & Therapy, 5(1), 30. [DOI:10.1186/scrt419] [PMID ] [PMCID ] 24572146 PMC4055132

[B21] English K. P Mahon B. J Wood K. (2014). Mesenchymal stromal cells: Role in tissue repair, drug discovery and immune modulation. Current Drug Delivery, 11(5), 561–71. [DOI:10.2174/1567201810999131125222440] [PMID ] 23517624

[B22] Fu L. Zhu L. Huang Y. Lee T. D. Forman S. J. Shih C. C. (2008). Derivation of neural stem cells from mesenchymal stem cells: Evidence for a bipotential stem cell population. Stem Cells and Development, 17(6), 1109–22. [DOI:10.1089/scd.2008.0068] [PMID ] [PMCID ] 18426339 PMC3189713

[B23] Gronthos S. Mankani M. Brahim J. Robey P. G. Shi S. (2000). Postnatal human Dental Pulp Stem Cells (DPSCs) in vitro and in vivo. Proceedings of the National Academy of Sciences, 97(25), 13625–30. [DOI:10.1073/pnas.240309797] [PMID ] [PMCID ] PMC17626 11087820

[B24] Haratizadeh S. Bojnordi M. N. Niapour A. Bakhtiari M. Hamidabadi H. G. (2016). [Improvement of neuroglial differentiation from human dental pulp stem cells using CSF (Persian)]. Journal of Mazandaran University of Medical Sciences, 26(140), 1–14.

[B25] Hung S. C. Cheng H. Pan C. Y. Tsai M. J. Kao L. S. Ma H. L. (2002). In vitro differentiation of size-sieved stem cells into electrically active neural cells. Stem Cells, 20(6), 522–9. [DOI:10.1634/stemcells.20-6-522] [PMID ] 12456960

[B26] Karaöz E. Demircan P. C. Sağlam Ö Aksoy A. Kaymaz F. Duruksu G. (2011). Human dental pulp stem cells demonstrate better neural and epithelial stem cell properties than bone marrow-derived mesenchymal stem cells. Histochemistry and Cell Biology, 136(4), 455–73. [DOI:10.1007/s00418-011-0858-3] [PMID ] 21879347

[B27] Karaöz E. Doğan B. N. Aksoy A. Gacar G. Akyüz S. Ayhan S. (2010). Isolation and in vitro characterisation of dental pulp stem cells from natal teeth. Histochemistry and Cell Biology, 133(1), 95–112. [DOI:10.1007/s00418-009-0646-5] [PMID ] 19816704

[B28] Király M. Kádár K. Horváthy D. B. Nardai P. Rácz G. Z. Lacza Z. (2011). Integration of neuronally predifferentiated human dental pulp stem cells into rat brain in vivo. Neurochemistry International, 59(3), 371–81. [DOI:10.1016/j.neuint.2011.01.006] [PMID ] 21219952

[B29] Kiraly M. Porcsalmy B. Pataki A. Kadar K. Jelitai M. Molnar B. (2009). Simultaneous PKC and cAMP activation induces differentiation of human dental pulp stem cells into functionally active neurons. Neurochemistry International, 55(5), 323–32. [DOI:10.1016/j.neuint.2009.03.017] [PMID ] 19576521

[B30] Kitamura C. Shirai K. Inoue M. Tashiro T. (2007). Changes in the subcellular distribution of microtubule-associated protein 1B during synaptogenesis of cultured rat cortical neurons. Cellular and Molecular Neurobiology, 27(1), 57–73. [DOI:10.1007/s10571-006-9117-x] [PMID ] 17151949 PMC11517391

[B31] Lariviere R. C. Julien J. P. (2004). Functions of intermediate filaments in neuronal development and disease. Developmental Neurobiology, 58(1), 131–48. [DOI:10.1002/neu.10270] [PMID ] 14598376

[B32] Le Blanc K. (2006). Mesenchymal stromal cells: Tissue repair and immune modulation. Cytotherapy, 8(6), 559–61. [DOI:10.1080/14653240601045399] [PMID ] 17148032

[B33] Lee H. J. Kim K. S. Ahn J. Bae H. M. Lim I. Kim S. U. (2014). Human motor neurons generated from neural stem cells delay clinical onset and prolong life in ALS mouse model. PloS One, 9(5), e97518. [DOI:10.1371/journal.pone.0097518] [PMID ] [PMCID ] PMC4028267 24844281

[B34] Leong W. K. Henshall T. L. Arthur A. Kremer K. L. Lewis M. D. Helps S. C. (2012). Human adult dental pulp stem cells enhance post stroke functional recovery through non-neural replacement mechanisms. Stem Cells Translational Medicine, 1(3), 177–87. [DOI:10.5966/sctm.2011-0039] [PMID ] [PMCID ] 23197777 PMC3659845

[B35] Lu P. Blesch A. Tuszynski M. H. (2004). Induction of bone marrow stromal cells to neurons: Differentiation, transdifferentiation, or artifact. Journal of Neuroscience Research, 77(2), 174–91. [DOI:10.1002/jnr.20148] [PMID ] 15211585

[B36] Mead B. Logan A. Berry M. Leadbeater W. Scheven B. A. (2014). Paracrine-mediated neuroprotection and neuritogenesis of axotomised retinal ganglion cells by human dental pulp stem cells: Comparison with human bone marrow and adipose-derived mesenchymal stem cells. PLoS One, 9(10), e109305. [DOI:10.1371/journal.pone.0109305] [PMID ] [PMCID ] PMC4188599 25290916

[B37] Moayeri A. Bojnordi M. N. Haratizadeh S. Esmaeilnejad-Moghadam A. Alizadeh R. Hamidabadi H. G. (2017). Transdifferentiation of human dental pulp stem cells into oligoprogenitor cells. Basic and Clinical Neuroscience, 8(5), 387–94. [DOI:10.18869/nirp.bcn.8.5.387] [PMID ] [PMCID ] 29167725 PMC5691170

[B38] Naghdi M. Tiraihi T. Mesbah-Namin S. A. Arabkharadmand J. Kazemi H. Taheri T. (2013). Improvement of contused spinal cord in rats by cholinergic-like neuron therapy. Iranian Red Crescent Medical Journal, 15(2), 127–35. [DOI:10.5812/ircmj.7653] [PMID ] [PMCID ] 23682324 PMC3652499

[B39] Naghdi M. Tiraihi T. Namin S. A. M. Arabkheradmand J. (2009). Transdifferentiation of bone marrow stromal cells into cholinergic neuronal phenotype: a potential source for cell therapy in spinal cord injury. Cytotherapy, 11(2), 137–52. [DOI:10.1080/14653240802716582] [PMID ] 19253075

[B40] Ni L. Wen Y. Peng X. Jonakait G. M. (2001). Antioxidants N-acetylcysteine (NAC) and 2-mercaptoethanol (2-ME) affect the survival and differentiative potential of cholinergic precursors from the embryonic septal nuclei and basal forebrain: Involvement of RAS signaling. Developmental Brain Research, 130(2), 207–16. [DOI:10.1016/S0165-3806(01)00238-3] 11675123

[B41] Nizzardo M. Simone C. Falcone M. Locatelli F. Riboldi G. Comi G. P. (2010). Human motor neuron generation from embryonic stem cells and induced pluripotent stem cells. Cellular and Molecular Life Sciences, 67(22), 3837–47. [DOI:10.1007/s00018-010-0463-y] [PMID ] 20668908 PMC11115886

[B42] Nosrat I. V. Smith C. A. Mullally P. Olson L. Nosrat C. A. (2004). Dental pulp cells provide neurotrophic support for dopaminergic neurons and differentiate into neurons in vitro: Implications for tissue engineering and repair in the nervous system. European Journal of Neuroscience, 19(9), 2388–98. [DOI:10.1111/j.0953-816X.2004.03314.x] [PMID ] 15128393

[B43] Ronaghi M. Erceg S. Moreno-Manzano V. Stojkovic M. (2010). Challenges of stem cell therapy for spinal cord injury: human embryonic stem cells, endogenous neural stem cells, or induced pluripotent stem cells. Stem Cells, 28(1), 93–9. [DOI:10.1002/stem.253] [PMID ] 19904738

[B44] Sakai K. Yamamoto A. Matsubara K. Nakamura S. Naruse M. Yamagata M. (2012). Human dental pulp-derived stem cells promote locomotor recovery after complete transection of the rat spinal cord by multiple neuro-regenerative mechanisms. The Journal of Clinical Investigation, 122(1), 80–90. [DOI:10.1172/JCI59251] [PMID ] [PMCID ] 22133879 PMC3248299

[B45] Sheng M. (2001). Molecular organization of the postsynaptic specialization. Proceedings of the National Academy of Sciences, 98(13), 7058–61. [DOI:10.1073/pnas.111146298] [PMID ] [PMCID ] PMC34622 11416187

[B46] Shi S. Robey P. G. Gronthos S. (2001). Comparison of human dental pulp and bone marrow stromal stem cells by cDNA microarray analysis. Bone, 29(6), 532–9. [DOI:10.1016/S8756-3282(01)00612-3] 11728923

[B47] Soundararajan P. Lindsey B. W. Leopold C. Rafuse V. F. (2007). Easy and rapid differentiation of embryonic stem cells into functional motoneurons using sonic hedgehog-producing cells. Stem Cells, 25(7), 1697–706. [DOI:10.1634/stem-cells.2006-0654] [PMID ] 17395777

[B48] Takahashi J. Palmer T.D. Gage F.H., (1999). Retinoic acid and neurotrophins collaborate to regulate neurogenesis in adult-derived neural stem cell cultures. Developmental Neurobiology, 38(1), 65–81. [DOI:10.1002/(SICI)1097-4695(199901)38:13.0.CO;2-Q] 10027563

[B49] Thonhoff J. R. Ojeda L. Wu P. (2009). Stem cell-derived motor neurons: applications and challenges in amyotrophic lateral sclerosis. Current Stem Cell Research & Therapy, 4(3), 178–99. [DOI:10.2174/157488809789057392] [PMID ] 19492980 PMC2887342

[B50] Wichterle H. Lieberam I. Porter J. A. Jessell T. M., (2002). Directed differentiation of embryonic stem cells into motor neurons. Cell, 110(3), 385–97. [DOI:10.1016/S0092-8674(02)00835-8] 12176325

